# Air quality and mental health: evidence, challenges and future directions

**DOI:** 10.1192/bjo.2023.507

**Published:** 2023-07-05

**Authors:** Kamaldeep Bhui, Joanne B. Newbury, Rachel M. Latham, Marcella Ucci, Zaheer A. Nasir, Briony Turner, Catherine O'Leary, Helen L. Fisher, Emma Marczylo, Philippa Douglas, Stephen Stansfeld, Simon K. Jackson, Sean Tyrrel, Andrey Rzhetsky, Rob Kinnersley, Prashant Kumar, Caroline Duchaine, Frederic Coulon

**Affiliations:** Department of Psychiatry, University of Oxford, UK; Nuffield Department of Primary Care Health Sciences, Medical Sciences Division, Wadham College, University of Oxford, UK; World Psychiatric Association Collaborating Centre, UK; Oxford Health NHS Foundation Trust, UK; East London Foundation NHS Trust, UK; and Oxford Health NIHR Biomedical Research Centre, UK; Centre for Academic Mental Health, Population Health Sciences, Bristol Medical School, University of Bristol, UK; and MRC Integrative Epidemiology Unit, Population Health Sciences, Bristol Medical School, University of Bristol, UK; Social, Genetic & Developmental Psychiatry Centre, Institute of Psychiatry, Psychology and Neuroscience, King's College London, UK; and ESRC Centre for Society and Mental Health, King's College London, UK; UCL Institute for Environmental Design and Engineering, University College London, UK; School of Water, Energy and Environment, Cranfield University, UK; National Centre for Earth Observation, Department of Meteorology, University of Reading, UK; Radiation, Chemical and Environmental Hazards, UK Health Security Agency, UK; and Centre for Environmental Health and Sustainability, University of Leicester, UK; Radiation, Chemical and Environmental Hazards, UK Health Security Agency, UK; Environment Agency, UK; Chief Scientist's Group, Environment Agency, UK; and Centre for Environmental Health and Sustainability, University of Leicester, UK; Centre for Psychiatry, Barts & The London School of Medicine and Dentistry, Queen Mary University of London, UK; School of Biomedical Science, Faculty of Health Sciences, University of Plymouth, UK; Department of Medicine, The University of Chicago, USA; Department of Human Genetics, The University of Chicago, USA; and Institute for Genomics and Systems Biology, The University of Chicago, USA; Chief Scientist's Group, Environmental Agency, UK; Global Centre for Clean Air Research (GCARE), School of Sustainability, Civil and Environmental Engineering, Faculty of Engineering and Physical Sciences, University of Surrey, UK; Department of Biochemistry, Microbiology and Bioinformatics, Université Laval, Canada; and Quebec Heart and Lung Institute, Université Laval, Canada

**Keywords:** Air quality, pollution, research, policy, mental health

## Abstract

**Background:**

Poor air quality is associated with poor health. Little attention is given to the complex array of environmental exposures and air pollutants that affect mental health during the life course.

**Aims:**

We gather interdisciplinary expertise and knowledge across the air pollution and mental health fields. We seek to propose future research priorities and how to address them.

**Method:**

Through a rapid narrative review, we summarise the key scientific findings, knowledge gaps and methodological challenges.

**Results:**

There is emerging evidence of associations between poor air quality, both indoors and outdoors, and poor mental health more generally, as well as specific mental disorders. Furthermore, pre-existing long-term conditions appear to deteriorate, requiring more healthcare. Evidence of critical periods for exposure among children and adolescents highlights the need for more longitudinal data as the basis of early preventive actions and policies. Particulate matter, including bioaerosols, are implicated, but form part of a complex exposome influenced by geography, deprivation, socioeconomic conditions and biological and individual vulnerabilities. Critical knowledge gaps need to be addressed to design interventions for mitigation and prevention, reflecting ever-changing sources of air pollution. The evidence base can inform and motivate multi-sector and interdisciplinary efforts of researchers, practitioners, policy makers, industry, community groups and campaigners to take informed action.

**Conclusions:**

There are knowledge gaps and a need for more research, for example, around bioaerosols exposure, indoor and outdoor pollution, urban design and impact on mental health over the life course.

## Aim, scope and methodological approach

The purpose of this rapid narrative review is to gather expert opinions and summarise the existing body of knowledge on air quality and the long-term effects on mental health, highlight methodological challenges and knowledge gaps and identify future research directions. The perspective we take is broad, interdisciplinary and adopts a ‘life-course’ approach, considering psychiatric, cognitive and neurodevelopmental pathways and a wide spectrum of both indoor and outdoor air pollutants, including bioaerosols, heavy metal ions, inorganic particulate matter (PM) and gaseous pollutants.

Existing reviews have mostly focused on associations between air pollution and one type of mental health problem, using multiple study designs. For example, an excellent recent systematic review shows convincing evidence of associations between depression and PM_2.5_.^1^ This included five cohort studies and mostly cross-sectional and time-series studies from high- and low-income countries; the authors report significant heterogeneity and potential selection biases, but find convincing evidence of links between particulate matter and depression. There is much less research on psychoses, and specific conditions such as schizophrenia or personality disorders. One review argues cogently that exposure to xenobiotic heavy metals (such as lead and cadmium), particulate matter and nitrogen and sulphur oxides, organic solvents and other constituents of environmental pollution could be component causes of neurodevelopmental disorders such as schizophrenia.^2^

The work is undertaken by BioAirNet, a network funded by the UK Research & Innovation (UKRI) agency, bringing together diverse disciplines to advance research, practice and policy. We aimed to provide an umbrella review including multiple mental problems, and the broadest range of literature in a very complex field, which necessarily brings together multiple disciplinary perspectives and contradictory positions. Although narrative reviews can undertake systematic searches, for complex and interdisciplinary narrative reviews, where the evidence is scattered across disciplinary journal, snowballing is considered a more appropriate approach and yields a fuller body of evidence.^3,4^ Even when systematically analysed, the conclusions of conventional reviews for complex areas often suggest there is inadequate evidence to draw firm conclusions. Consequently, the BioAirNet team identified suitable literature through their existing work and networks. We added our interdisciplinary dialogue through workshops as an additional source of synthesis. This is a critical step, as assumed knowledge in one discipline is not necessarily that in another; furthermore, epistemic processes in each discipline and across academic–community partnerships often lead to some knowledge being valued and some being dismissed.^5,6^ Hence, in BioAirNet and related UKRI-funded air quality networks, our desire is to establish a cross-disciplinary scaffolding and opportunity for progressing research optimally so it can have a greater impact on public health, offering a starting point and foundation for future research efforts.

We present our review findings by examining the health burden of air pollution and the influence of outdoor and indoor environments; outdoor and indoor air pollution and mental health; and the impact of air pollution on mental health over the life course, from pregnancy, through childhood, to adult and older populations. We also consider research methodological challenges.

Then, we present knowledge gaps and recommendations for research emerging from the literature and from our early workshops. Finally, of all the possible studies, we propose priority research topics and related methods, taking account of the earlier learning from the reviewed literature and workshops.

## The health burden of air pollution

The World Health Organization (WHO) has ranked air pollution as one of the major environmental health risks, and the single biggest environmental threat to human health.^7^ Worldwide, it is estimated that 4.2 million and 3.8 million premature deaths were attributable to outdoor and indoor air pollution, respectively.^7^ There is more evidence of the adverse health effects of particulate matter.^8^ Particulate matter has diverse sources (natural/anthropogenic, indoor/outdoor), formation processes, composition (organic/inorganic) and sizes (ultrafine: PM_0.1_, particles that are <0.1 μm in diameter; fine: PM_2.5_, particles that are <2.5 μm in diameter; coarse: particles that are >PM_2.5_ and <PM_10_ in diameter).

The WHO guidelines implicate particulate matter with aerodynamic diameters of ≤2.5 μm (PM_2.5_) and ≤10 μm (PM_10_), ozone, nitrogen dioxide, sulphur dioxide and carbon monoxide in poor air quality. The particle size can influence whether particulate matter can cross the blood–brain barrier and, along with duration of exposure, increase the risk of adverse health effects. Smaller particles are inhaled more deeply into the lung, leading to greater effects on health. The strongest evidence for adverse effects on health is for PM_2.5_, with an extensive body of evidence linking outdoor PM_2.5_ exposure to mortality, cardiovascular diseases, pulmonary diseases and cancer.^8,9^ Therefore, modifying exposure to poor air quality in indoor and outdoor environments could reduce the population-level burden of poor health.

### Outdoor air pollution

Outdoor air pollution, particularly particulate matter, is classified by the International Agency for Research on Cancer as carcinogenic to humans (a Group 1 carcinogen) and causes lung cancer.^10^ Given the high levels of incident serious mental illness in urban areas where air pollution is greatest, and reverse causal relationships between cancer and serious mental illness (see the section ‘Air quality and mental health over the life course'), there may be common aetiological and mutually reinforcing pathways of risk involving air pollution and inflammation,^11^ and oncogenic impacts.^12^

Bioaerosols are the biological fraction of particulate matter and are a complex mixture of bacteria, viruses and fungi, or parts of living organisms, like pollen, spores, endotoxins from bacterial cells and mycotoxins from fungi.^13,14^ Bioaerosol exposure is associated with chronic and acute respiratory illness (via both atopic and non-atopic allergic mechanisms, and non-allergic pathways like infection), and other diseases including gastrointestinal disturbance, dermatological conditions, general malaise and fatigue.^13,14^ However, the role of biological particulate matter in health burden, their mechanisms of toxicity and impact on human health and well-being across the indoor–outdoor continuum of exposure is not yet clear. Conclusive evidence linking the exact mode of action between pollution, including bioaerosol exposure and its related toxicity, is lacking. However, airway inflammation and oxidative stress are recognised as major mechanisms of the diseases because of particulate matter and associated microbe exposure.^15,16^ In particular, bacterial endotoxin (lipopolysaccharides) and fungi are linked with inflammatory responses and hypersensitivity in airway models.^16,17^

Inflammation is implicated in pathways to poor health, for which the emerging evidence is credible for both mental and physical conditions.^18–20^ The exact process by which inflammation (peripheral and brain tissue) leads to neurotoxic effects is dynamic, complex and subject to numerous self-regulatory processes;^21^ for example, internalising symptoms of anxiety and depression, fronto-limbic brain areas responsible for emotional regulation, and neuroinflammation and oxidative stress are implicated.^22,23^ In a systematic review by Zundel et al, air pollution was consistently associated with neurostructural and neurofunctional effects such as inflammation and oxidative stress, changes to neurotransmitters, neuromodulators and their metabolites, within multiple brain regions (24% of papers), the hippocampus (66%), prefrontal cortex (7%) and amygdala (1%).^22^

Shared inflammatory mechanisms of disease aetiology, if confirmed, offer hope for new forms of prevention and treatment that target inflammation by repurposing well-established and relatively safe anti-inflammatory drugs.^24^ Furthermore, in children exposed to fine and ultrafine particulate matter, there is evidence of the hallmarks of Alzheimer's and Parkinson's diseases, namely hyperphosphorylated tau, amyloid plaques and misfolded α-synuclein.^23^ There is emerging evidence of air pollution in cognitive function and dementia.^25^ Brain imaging and animal studies could help to further elucidate relevant mechanisms. A recent systematic review suggests that depression, suicide and neurodevelopmental disorders (such as autism for pregnancy-related exposures) may be more common among those exposed to air pollution.^26^

### Urban design and indoor environments

The effect of outdoor air quality on indoor exposures is a controversial area, with contradictory findings.^27^ Research follows two main lines, either assuming linear correlations between indoor and outdoor pollution, or the need for measurement of dynamic indoor and outdoor pollution ratios.^27^ External levels of bioaerosols and particulate matter influence indoor levels, yet there may be additional indoor sources. Indoor air quality can be poorer because of ventilation systems drawing in highly polluted air; for example, from diesel fumes from lorries parked near vents. Concentrations of pollutants are worse where residents are smokers, use open fires for warming the house and where cooking fats and emissions are not cleared.

**Fig. 1:**
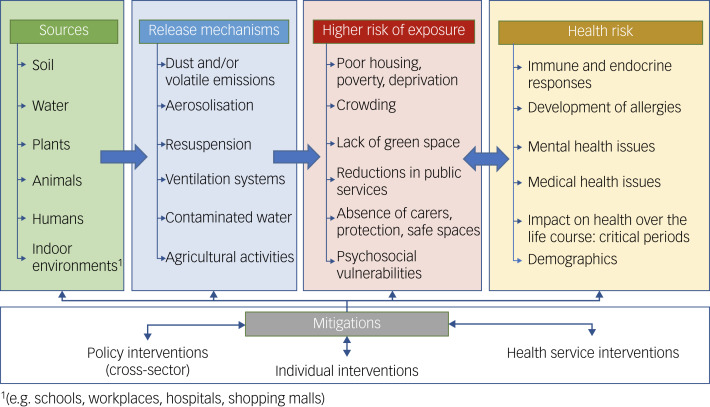
Complex webs of causation linking air quality with health.

Re-design of environments and buildings may have several benefits.^28^ In areas of high deprivation and urbanicity, several co-occurring risk factors are common: poverty and lack of affordable housing, unemployment and lack of green space, and unsafe neighbourhoods. These multiple and chronic adversities are associated with inflammation and, through interactions with air quality, can lead to more physical and mental ill health (see Fig. 1).^29,30^

## Mental health and air pollution

**Fig. 2:**
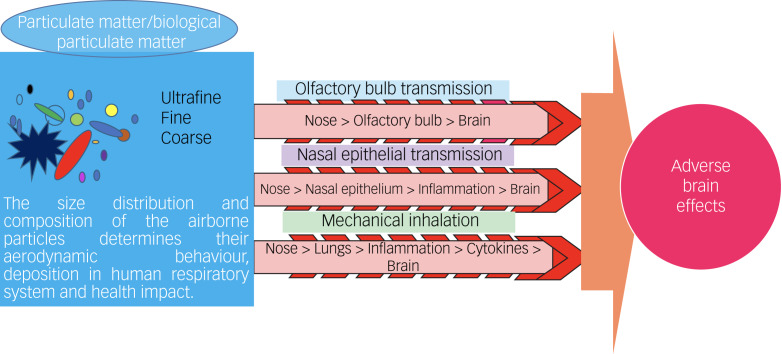
Potential pathways from particulate matter/biological particulate matter to adverse effects on brain health.

Alongside effects on cardiovascular and respiratory health, there is emerging evidence that exposure to air pollutants (both indoors and outdoors) may lead to neurocognitive disorders and affect mental health (directly and indirectly) through a range of potential causal pathways (see Figs 1 and 2).^1,31–35^ Observational evidence has implicated outdoor air pollutants as risk factors for a variety of mental health problems, including depression, anxiety, personality disorders and schizophrenia.^36–40^ In contrast, there is less research on the effects of indoor air quality and exposures to air pollutants on mental health. Yet, some aspects will be common; for example, inadequate housing is more common in urban spaces, where outdoor and indoor air quality is poorer.

### Outdoor air quality

Much of the existing literature on outdoor air pollution and mental health is based on cross-sectional observations, aggregated air quality data and studies performed only in adults. In addition, studies often do not rule out alternative interpretations, such as individuals with a greater liability to mental health problems self-selecting into neighbourhoods with poorer air quality or not being able to leave those areas. Furthermore, other disadvantageous aspects of the environment are also associated with poor air quality and poor mental health; these include deprivation, crime and noise, which each might affect mental health. However, to understand the causal role of air pollution in the development of mental health problems, longitudinal studies are needed to ensure exposure to air pollution does occur before the emergence of mental health problems. Ideally, these studies should collect information on individual- and neighbourhood-level deprivation. Causal inferences are strengthened by such designs, although confounding influences do still need consideration.

### Indoor environments

Since a great proportion of time is spent indoors, especially in the winter and autumn months, it is reasonable to assume that some of the effects attributed to outdoor air pollutants result from indoor exposures.^41^ Indoor environments can have diverse pollutants (e.g. particulate matter, nitrogen dioxide, carbon monoxide) of outdoor and indoor origin, and highly varying source strength for each pollutant across different indoor environments. Yet indoor environments may also be a more significant source of specific chemical exposures (e.g. volatile and semi-volatile organic compounds). Much built environment research is model based, with less real-world sampling in diverse geographical contexts. The health evidence often lacks housing and geolocation information, making it difficult to review historical data for causal trends retrospectively. Both types of approach would benefit from greater interaction with chemists so that we can better understand pollutants, and potential webs of causation. For example, the impact of cooking emissions on human and environmental health can be reduced by better-designed research that might help to reconsider open-plan kitchen and living spaces.^42^ Different factors related to design, construction and occupants’ activities can help to determine occupants’ exposure to different pollutants indoors.^43^ Additionally, the growing focus on energy-efficient built environments may lead to increased exposure to an array of air pollutants of indoor origin, because of the decreased ventilation and potential increase in pollutant concentrations.^28,44^ Sound insulation may also reduce ventilation and more heat efficient, but raise indoor temperatures that influence the composition of particulate matter; hence, there is a need for building designs that tackle multiple environmental factors. These design issues may explain low-grade fatigue and poor mental health found in certain home and work environments that lack ventilation, daylight and good air quality; ‘sick building syndrome’ may be partially explained by air quality.^45,46^

The Royal College of Paediatrics and Child Health and the Royal College of Physicians considered indoor air quality and found that emissions from construction materials, building design (e.g. ventilation and heating systems) and activities inside buildings (e.g. cooking, fireplaces, cleaning products, moisture production) all affect indoor air quality and affect health.^47^ Some activities can lead to elevated moisture levels indoors, resulting in dampness and related pollutants such as mould and house dust mites, which in turn affect health. Although this report did not specifically consider mental health outcomes, the underpinning studies found links between poor indoor air quality and neurological and psychological symptoms, with cognitive and behavioural effects. For example, higher carbon dioxide levels and indoor air pollutants associated with carbon dioxide, can negatively affect cognitive function and concentration. Of course, other indoor air pollutants that accumulate with carbon dioxide may be partially responsible. There is ongoing research and debate on the effects of carbon dioxide concentrations on cognitive performance in settings such as schools and offices.^48,49^ Some researchers have emphasised that fossil fuel combustion ‘is driving indoor CO_2_ [carbon dioxide] towards levels harmful to human cognition’,^50^ and that associations between cognitive performance and indoor levels of carbon dioxide, and volatile organic compounds, are independent of ventilation rates.^49^ A recent systematic review raises significant questions about the quality of this evidence, and whether any associations between carbon dioxide and health can yet be inferred, although several of the reviewed studies suggested that high concentrations of carbon dioxide was associated with increased mental effort and fatigue.^51^

Although, there is limited evidence on the impact of indoor air quality on mental health, some studies have found an association between depression and dampness and mould in the home; this tentatively suggested that a lack of control over the home environment was a potential mechanism leading to poor health.^52^ Furthermore, poverty and cockroach infestations are associated with elevated levels of endotoxins, also leading to inflammatory responses.^53^ A report by Shelter, a non-governmental organisation in the UK, concluded that 26% of households complain of significant dampness, mould and condensation.^54^ A recent government analysis following the death of a child because of damp conditions (coroner's verdict) estimates that 4% of social housing has notable damp and mould, and 0.2% has the most serious conditions that would fail the safe home standards.^55^ Associations between mould exposure and various non-specific symptoms such as fatigue, ‘brain fog’ and anxiety have been reported. Yet, overall, the evidence is mixed, and underlying mechanisms are not clear. More recently, studies using animal models suggested that mould inhalation affects the central nervous system and immune activation, with concomitant neural effects and cognitive, emotional and behavioural symptoms.^56,57^ Furthermore, flame retardants and plasticisers are common in indoor environments, and can cause adverse neurological effects and negative behavioural outcomes, including impaired learning and spatial memory.^58,59^

Poor socioeconomic status is known to be associated with both poor mental health and poor living conditions, including overcrowding, unstable housing, dampness, poor nutrition and health risk behaviours such as smoking, alcohol use, substance misuse and adverse childhood experiences (e.g. poverty, loss events and neglect).^60,61^ Consistent with this evidence, are studies of exposures to poor indoor air quality by low- and high-income settings; in low-income settings, burning unclean and solid biomass fuels dominate as contributors to air pollution.^62^

All of these influences might combine to create a pro-inflammatory exposome that includes poor air quality and affects health – both in the onset of new illnesses as well as the compounding of existing disabilities for pre-existing illnesses. Linking the associations with mechanisms is challenging (see Figs 1 and 2 for potential explanations). Yet, there are plausible pathways from pollution to poor health and poor mental health, especially if shared aetiologies (e.g. inflammatory processes as one example) are triggered. One approach to understanding prevention and care is to look at the individual life course, from pregnancy to youth, adulthood and old age.

## Air quality and mental health over the life-course

### Pregnancy and early years

Studies of associations between early exposure to air pollution and mental health are scarce, and the findings are somewhat mixed. Prenatal air pollution exposure has been linked with cognitive impairments at 5 years of age,^63^ but there is no greater risk of anxiety and depressive symptoms.^64^ In a Spanish study of 1889 children, exposure to nitrogen dioxide and benzene were inversely associated with mental development, but this did not remain a statistically significant finding after adjusting for confounders.^65^ There are some details of relevance: stronger inverse associations were estimated for pollutants among infants whose mothers reported low intakes of fruits/vegetables during pregnancy, in non-breastfed infants and infants with low maternal vitamin D; however, these interesting interactions were not statistically significant. During pregnancy, exposure to PM_10_, PM_2.5_, nitrogen dioxide and nitrogen oxides were associated with a 29–74% increased odds of unspecified mental disorders that complicated pregnancy.^66^ Exposure pathways *in utero* and early childhood also differ from those in adulthood; for example, *in utero*, neo-natal and infancy-related pathways may include ingestion (non-nutritional as well as nutritional), inhalation, transplacental, transdermal and breastfeeding.^62^

### Adolescents

Prevention of mental illness early in the life course is critical, given that half of adults with mental illnesses show signs and symptoms by 11 years of age, and 75% do so by 24 years of age.^67,68^ In addition to the human suffering and functional impairment caused by chronic mental health problems, adults with mental illnesses face premature mortality of 15–20 years as a result of cancer, heart disease, lung disease and obesity-related conditions.^69–71^ Identifying modifiable risk factors for mental health problems is, therefore, a crucial research challenge of the 21st century. A long-standing finding that has not been fully explained is the higher incidence rates of psychoses in inner city and urban areas, and these usually arise during adolescence and early adulthood.^72,73^ Could air quality be a relevant aetiological factor? Among 2063 adolescents, psychotic experiences were significantly more common among adolescents with the highest (top quartile) level of annual exposure to nitrogen dioxide and PM_2.5_.^74^ Together, nitrogen dioxide and nitrogen oxides explained 60% of the variance. There was no evidence of confounding by family socioeconomic status, family psychiatric history, maternal psychosis, childhood psychotic symptoms, adolescent smoking and substance dependence. There is also evidence of associations with depression.^75^ In the Environmental Risk Longitudinal Twin Study of 2039 participants, after adjustment for family and individual factors, interquartile range increments in exposure to nitrogen oxides were associated with a 1.4-point increase in general psychopathology.^76^ There was no association between continuously measured PM_2.5_ and general psychopathology. However, those in the highest quartile of PM_2.5_ exposure scored higher than those in the bottom three quartiles. There were also statistically significant findings for nitrogen oxides. Exposure to nitrogen oxides was associated with all secondary outcomes, but associations were weakest for internalising and strongest for ‘thought disorder’, a symptom of psychosis. Studies to replicate this and evaluate the source of these differential effects are needed. Despite nitrogen oxide concentrations being highest in neighbourhoods with worse physical, social and economic conditions, adjusting estimates for neighbourhood characteristics did not change the results, suggesting other neighbourhood characteristics may be driving the associations.

### Adults

A study in the USA and Denmark assessed air pollution on an air quality index of 87 potential air pollutants in the USA and 14 in Denmark. PM_10_ and PM_2.5_, diesel emissions, nitrogen dioxide and organic substances (such as polycyclic aromatic hydrocarbons) were significantly associated with an increased risk of psychiatric disorders.^77^ The country-specific data showed pollution exposure to be associated with bipolar disorder in both countries, and depression, schizophrenia and personality disorder in Denmark. A number of studies show associations between air pollution and service use for mental disorders.^31,78–80^

A recent systematic review and meta-analysis showed associations of PM_2.5_ and PM_10_ with depression, anxiety, bipolar disorder, psychosis and suicide in adults. The most apparent association was between long-term (>6 months) exposure to PM_2.5_ and depression.^81^ Depression and suicide were the most studied outcomes; however, there were no studies of long-term particulate matter exposure and suicide, nor of particulate matter exposure and bipolar disorder. The review highlighted a need for larger-scale longitudinal studies using representative samples, and adjustments for area-level factors such as traffic noise, access to green space and socioeconomic status, to help better understand potential causality in observed associations. Further research is needed on the mechanisms involved in these observed associations.

## Methodological issues in air pollution and mental health research

Outdoor air pollution can be measured and estimated in numerous ways.^82,83^ Here, we describe some of the main methods used in the field of air pollution and mental health, in roughly chronological order.

From Tuke in Victorian Britain, through to Durkheim, Jarvis, and Faris and Dunham in the 1930s, the social and geographical distribution of mental illnesses have been investigated so as to understand how urbanisation and rurality might affect mental health.^84^ The manner in which the social and ecological environment shape neurodevelopment and mental illness in early adolescence and adulthood are important, but are often neglected in favour of a more deterministic approach to understanding the causes of mental illnesses; rather, we propose place and contextual bodies of evidence are important, on which studies of air quality can naturally build.^85^ Furthermore, new studies must accommodate existing research on urban environments, including building design, and how neurodevelopment and social meaning interact.^86,87^

Dating back to the seminal work by Faris and Dunham (1939),^88^ a precursor to the air pollution and mental health field is the body of research demonstrating associations between the urban environment and mental health, often by using population density or urban–rural comparisons. Air pollution has been speculated as a potential driver of this relationship.^89^ However, cities are complex environments comprising multiple correlated risk factors that could affect mental health, making urbanicity only a crude proxy for air quality. Nevertheless, a series of comparative studies based in Mexico used a similar design, comparing Mexico City to less polluted areas. Among these, one post-mortem study^90^ compared prefrontal white matter between children and teenagers who had lived in Mexico City versus a less urbanised area, and found that ultrafine particulate matter was found in the brain cells of those from Mexico City, but not those from the less urbanised area.

Among the earliest studies are some exploring associations between people's perceptions of air pollution and mental health. For example, Evans et al^91^ asked residents of Los Angeles to rate the level of smog that day, from 1 (no smog) to 10 (heavy smog), and examined correlations of these responses with depression, anxiety and hostility. Although relatively economical to conduct and complete individual-level analysis, this type of design does not examine a direct biological effect of air pollution on the brain. In addition, this design is only suited to air pollutants that can be seen or smelled, which excludes many types such as carbon monoxide, which can have effects on neurological functions. Research on the self-reported perception of indoor air quality also shows that factors other than indoor pollutant concentrations can affect perceptions, such as occupational status or thermal sensation. Similar findings exist for environmental noise (in particular traffic noise)^92,93^ and various components of air pollution (in particular, particulate matter), which cluster with poor air quality.^94^ Thus, it is difficult to disentangle the effects of noise and air pollution. Noise can also increase the risk of mental disorders such as depression, anxiety disorders, psychoses and suicide.^95^ These place effects and other different potential causal factors need to be considered in future studies. Proximity to roads, for example, is also used as a proxy for air pollution exposure and noise.^96,97^ For instance, using data from the Danish Civil Registration System, Pedersen and Mortensen^97^ examined the association between distance to major roads and schizophrenia. The authors used official classifications of road types and geographic information system software to calculate the distance between households and the nearest major road. This innovative methodology still does not factor in other sources of air pollution, meteorological patterns or urban morphometric features (e.g. pockets of air pollution trapped between high-rise buildings).

One of the most common methods to measure air pollution concentrations directly at monitoring sites is to use passive diffusion tubes. Some studies have also set up monitoring stations at the locations of interest to measure real-time concentrations. For instance, Wang et al^98^ installed nitrogen dioxide and PM_10_ monitors at various locations within two schools in Quanzhou, China, and examined associations with neurologic functioning. The most common design is to use data from existing, permanent monitoring stations, often in conjunction with a time-series analysis design. For instance, Gu et al^99^ obtained data on daily average pollution concentrations for 75 Chinese cities from China's National Air Quality Monitoring System, and examined correlations with daily hospital admissions for depression. Although this time-series design is powerful in terms of understanding potential short-term effects of air pollution, monitoring stations are often very sparse, making it inappropriate to infer individual long-term exposure from the data.

The measurement techniques used for sampling outdoor pollution can also be used in indoor settings.^83^ Usually, measurements from static loggers and/or passive samplers placed within representative rooms and/or locations within a room are used as proxy of exposure to indoor pollutants. Some industry and International Organization for Standardization standards exist for the monitoring of specific indoor air pollutants.^100^ Pollutants such as carbon dioxide or total volatile organic compounds are sometimes used as a proxy of air quality and ventilation in indoor settings. Indoor concentrations can differ even when building layout/location is similar, because of variations in indoor sources and activities. Therefore, it is not always possible to deduce indoor pollution levels via limited sampling sites. Overall, monitoring indoor air quality at scale can be time-consuming and relatively expensive, requiring access to several properties/participants. On the other hand, low-cost sensing technologies also have the potential to provide high-density spatial–temporal information on air quality across the indoor–outdoor continuum, although accuracies can vary.^101,102^ Better assessment methods of indoor air quality with standardised and validated measures of built design may benefit from the expertise of engineers.^103^

Recently, more sophisticated methods of modelling outdoor air pollution concentrations have enabled much higher resolution estimates to be achieved, thereby facilitating more precise, individual-level exposure based on, for example, residential addresses. One dominant method is land-use regression modelling, which factors in environmental characteristics with predictable influences on pollution concentrations, such as road, factories and forests, to estimate pollution concentrations in a given area.^104^ Another method, called dispersion modelling, additionally factors in the atmospheric chemistry of air pollutants together with meteorological data, to estimate pollution concentrations.^105^ Dispersion models now achieve good predictions against ground-based measurements, as well as high temporal (e.g. hourly) and spatial (e.g. 20 m × 20 m) precision.^106^

The power of these models in understanding links between outdoor air quality and mental health lies in the ability to link this exposure data with large-scale epidemiological cohort studies. There are several important benefits of this large multidisciplinary consortium approach. First, the large sample sizes afford the statistical power to detect small effects, which may be needed in contexts (such as Europe and the USA) where pollution levels and variability are relatively low.^81^ Second, together with their large samples, the comprehensive assessment of a wide range of measures provides a valuable opportunity to adjust for multiple confounders and rule out threats to causal inference. These approaches can enable investigations of the role of important social and biological factors as mediators or moderators of associations, including psychosocial adversity, social deprivation, noise pollution, genetic risk and inflammation. Third, the prospective longitudinal design of cohort studies can help establish the temporality of associations, and therefore move the research on from reliance on cross-sectional observations that severely limit causal inference. Fourth, depending on the age and duration of the particular cohort, the impact of outdoor air pollutants on mental health can be explored across developmental periods across the life span, and consider residential mobility and distinct geographical contexts.

There is a need for more research focused on early-life exposure as *in utero* and childhood may be a time of particular vulnerability because the lungs, brain and immune system are developing. Indeed, a focus on such early pollution exposure and its later effects is particularly necessary to elucidate its role in the development of mental health problems, given the common onset of symptoms in childhood and adolescence.^107^ In the UK, birth cohort studies such as the Avon Longitudinal Study of Parents and Children (ALSPAC) and Environmental Risk (E-Risk) Longitudinal Twin Study have linked high-resolution air pollution models to their data.^74,76,108–110^ Combining epidemiological approaches with air pollution modelling in this way has yielded important insights. However, this methodology is not without limitations. Exposure estimates are modelled rather than measured directly. Typically, these are linked to just one or a few addresses commonly visited by the study participants (e.g. home, school/college, shops). To better quantify levels of air pollution exposure, multiple different locations are needed, as well as several different time points.

Although some data on indoor/outdoor ratios exist for some pollutants, indoor concentrations are not solely driven by outdoor levels. Therefore, air pollution modelling of outdoor levels could be combined with indoor modelling (or monitoring), to better understand patterns of exposure, and provide more representative exposure estimates accounting for the time spent indoors versus outdoors. Various approaches to modelling indoor air quality exist, including mass balance or computational fluid dynamic models.^100^ These can be used to estimate respective pollutant concentrations and their spatial distribution, and can be combined with meta-models of the building stock to estimate indoor air quality at scale.^111^ However, models of indoor air quality rely on assumptions about indoor sources and human behaviours, for which there are limited empirical data.

Wearable, personal monitoring devices that measure pollutant concentrations close to the person's breathing zone offer a promising alternative. These enable individuals’ exposure to be directly measured in real time as they go about their usual activity. As people spend time in and move between spaces with varying concentrations of pollution, these devices more accurately capture their unique exposure. Although currently expensive – prohibitively so for large samples – future studies should consider utilising new technologies that allow personal monitoring to move toward accurately capturing air pollution exposure in everyday life. The emergence of low-cost sensing technologies has the potential to provide high-density spatial–temporal information on air quality and personal exposure across indoor–outdoor continuum.^112,113^

## Research gaps and challenges

In the early phase of the BioAirNet (https://bioairnet.co.uk/) research network, a sandpit event involving multidisciplinary experts and a range of stakeholders was held. This sought to identify key research questions, knowledge gaps and methodological challenges. In combination with the literature we identified, the following priority research questions and knowledge gaps warrant future attention:

- Could air pollutant exposure and inflammatory mechanisms explain higher rates of mental illnesses (psychoses and affective disorders) in urban areas; variations of incident mental illnesses by age, gender, sexuality, ethnicity and deprivation; and greater risk for chronic health conditions into adulthood, including psychoses, common mental illnesses and comorbid medical conditions?
- What future environmental designs and practices (outdoor, indoor, buildings and institutions) might prevent and reduce the risks of poor health, especially in specific at-risk populations?
- How might specific interventions be developed and tested for impact on the mechanisms?
- How do we evaluate policy interventions and major policy re-designs, such as the introduction of low emission zone restrictions, which are being adopted in many cities?^114^
- Urban design evaluations also need methodologies that are feasible, adopting quasi-experimental designs, and collaboration between local government, building designer, epidemiologists/public health professionals, built environment architects and local residents.
- What constitutes an ‘anti-inflammatory’ environment that benefits young people in their worlds and adults at risk of or already experiencing mental illnesses and other health conditions?
- What role do social and behavioural factors have for creating or concentrating harmful exposomes in specific places and indoor environments, and mitigating these drivers of poor health?
- How do structural (socioeconomic, deprivation, poverty, geographical) and behavioural influences interact to promote or militate against a harmful exposome?
- How is child health and mental health affected, and what is the impact over the life course?
- How are specific high-risk groups affected: those with early psychoses, chronic depression and multimorbidity, including poor mental health?
- What are the implications for care environments for children and for those with mental illness?

In addition, specific approaches were identified to better quantify levels of exposure to indoor/outdoor pollution and links with impact on health in different scenarios (see Appendix 1); approaches to understand the mechanisms of harm to human health and well-being (see Appendix 2); and the need to specify more carefully which health conditions and causal models were being investigated (see Appendix 3).

These research gaps are broadly aligned with the six priorities proposed in a recent review of Environmental Science and Mental Health, including over 200 publications and six case studies.^115^ In this report, five areas of opportunity were identified, which consider both the research approach and topics warranting further investigation: exploit large-scale data-sets, longitudinal approaches, integrative complex systems research, mixed-methods approach and community of practice.

## Research design for a way forward

These priority research topics require advances in complex systems and mixed-methods research, and more capability to collect, analyse and use new data for policy actions. Research in this area needs to be interdisciplinary, and the methodologies selected will also need careful co-design and review to address the full range of research questions and knowledge gaps. The following section considers the potential study designs and recruitment venues for interdisciplinary research with health outcomes.

### School studies

Experience-based sampling is possible through mobile phone applications and wearable devices or by websites and self-report measures. The volume of data would not be sufficiently high, perhaps compared with school-based studies where young people usually complete the questionnaires in classes. A whole-school approach to support studies will be needed to ensure data quality, engagement and participation, and to ensure the research process itself is of value and beneficial and aligned with other priorities in schools.

Establishing partnerships and rapport with schools, higher education institutions and other stakeholders alongside developing appropriate teacher, community and parent panels, will support recruitment into studies and offer information about the acceptability of potential interventions and policy options.

The balance between entire school surveys and recruitment of young people experiencing specific conditions or vulnerabilities needs some debate; there are tensions in terms of acceptability, the ethical process for recruitment and consent, concerns about stigma and confidentiality, and methodological challenges of screening people into specific studies. A whole-school approach would permit a series of nested case–control studies for specific conditions and contexts.

Research studies will need appropriate ethical and safeguarding frameworks, especially for young people, but generally for any proposed intervention studies.

Some young people will not be in school, or will have been excluded, perhaps directly because of health problems and linked with adverse social circumstances that are likely to affect their health status; these groups may well be those most likely to be exposed to poor air quality. Thus, additional samples of excluded groups will need to be considered, alongside creative and innovative methods for including them. There are likely to be age-, gender-, sexuality- and ethnicity-related intersectional forces that are associated with exclusion and poor mental health. Specific consultation and sampling strategies will need to be devised.

### Longitudinal cohort studies with linkage

There are a number of existing cohorts that can be linked to data on air pollution. These offer opportunities to measure pollutants and mental health outcomes at multiple time points, resolving the temporal ordering and identifying critical periods for exposures and specific outcomes; the approach can also identify variation across multiple venues, and test generalisability and causal effects where exposures vary by geography. The linkage process takes time, yet some research groups have successfully done so and are generating new evidence in real time and gathering evidence on the entire exposome (e.g. the Equal-Life project, a European Union-funded programme; grant number 874724).^116^ The alternative approach is to design and establish new cohorts, with appropriate measures of air quality as well as the total hypothesised exposome.

### In-depth qualitative cohorts

This study design may be especially suited to exploring complex social, psychological and spatial mechanisms, generating new hypotheses and in-depth information about contexts and health status. Realist methodologies and ethnographies, for example, may reveal context, mechanism and outcome relationships, which can be tested in epidemiological cohorts. The approach is also suited to recruiting those at risk of not being represented in surveys and population, school-based and cohort studies. Obviously, this approach may not help identify or verify biological mechanisms unless biodata are collected alongside it. Such data could include functional brain scans, inflammatory markers, epigenetic effects and genetic liability through polygenic risk scores.

## Future directions and challenges

Air pollution and mental health are both major challenges that the world must grapple with, now and for years to come. This makes their intersection a doubly vital public health priority. This paper outlines evidence on the importance of indoor and outdoor air quality on mental health, research needs, challenges and future directions. There remain methodological challenges that must be overcome to provide insights into critical time points; place-based hot-spots for poor air quality; biological, psychological and social mechanisms; and strategies for prevention and mitigation. The clinical, public health and societal (well-being and economic) effects need to be modelled. Better quality primary research and longitudinal cohorts, especially for young people at critical points of maturation, are needed, alongside well-specified systematic reviews and network analyses. Specifically, areas of focus include evidence of links between pollution, specifically bioaerosols, and mental health, and better exposure measurement. An important linked but distinct subject that we have not addressed is climate change. The pathways between global warming, poor air quality, climate change and poor mental health may be mediated through natural disasters and social disruption, biodiversity loss and ecosystem destruction.^62^ Furthermore, there is little data on the contrasting effects of climate change on low- versus high-income countries. We know natural disasters and climate change will have more of an effect on low-income countries and poorer populations living in countries with less infrastructure, appropriate building design and protections around health and environmental policy.^117^ Furthermore, rising global temperatures are associated with more air pollution,^118^ including stagnation and less ventilation, and greater production of particulate matter (wildfire smoke, airborne soil dust, ozone and PM_2.5_). These affect long-term medical conditions (heart, lung and kidney disease) via raised body temperature and inflammation. Heat also leads to more anxiety and depressive symptoms, and suicidal behaviours; those with pre-existing mental illnesses are more likely to die in hot spells than those without mental illnesses.^119^ Engagement of policy stakeholders from diverse sectors is necessary to translate emergent findings into actions. We anticipate this paper and related publications from a number of networks will help bridge knowledge gaps to stimulate a new wave of research, practice and policy actions. This will enable us to gain deeper understanding of the intricate and interconnected relationship among individuals, air pollution exposure and resulting mental health and well-being effects, amid the continuously changing air pollution sources and exposure patterns. Ultimately, the knowledge gained should be used to inform policies concerning air pollution interventions, urban and built environment design, land use planning and behaviour change.

## Data Availability

Data availability is not applicable to this article as no new data were created or analysed in this study.

## Appendix 1

BioAirNet organised a series of thematic workshops focused on exploring the potential effects of biological particulate matter on human health, behaviour and well-being. Information on these workshops can be found on the BioAirNet website at https://bioairnet.co.uk/past-events/. During the workshops, lead researchers delved into the associations between biological particulate matter and respiratory and neurological disorders, and examined the approaches that can be used to understand the biological mechanisms underlying these associations. On average, over 30 delegates attended each workshop, indicating a significant interest and engagement with the topics. Because of the General Data Protection Regulation, we cannot disclose the names of individuals who attended these workshops publicly. Nevertheless, BioAirNet remains committed to involving participants from diverse backgrounds and interests in advancing our understanding of the factors that shape and support healthy natural and built environments, with a key focus on biological particulate matter. The Network is open to new members and collaborations. The following priorities were identified:

- Data-sets – matching (homogeneous) longitudinal exposure and health data-sets with comprehensive metadata for confounders;
- Models of exposure – personal versus population;
- New technologies (e.g. real-time pollution measurement, high-throughput sequencing, biosensors, personal devices) to inform exposure assessment;
- Proxy/biomarkers of exposure to inform exposure assessment;
- Statistical methods and study designs for linking exposure(s) to outcome(s) with appropriate power;
- Methods of measuring and understanding: the human microbiome and its role in inducing health outcomes; individual genetic diversity and potential impact on pollution-induced health outcomes; benefits versus harms, which particles are beneficial or harmful and in what contexts; background levels and exposure thresholds above which health is affected; vulnerable populations (e.g. by age, respiratory disease).

## Appendix 2

Study designs for future research:

- Literature reviews – linking exposure(s) to outcome(s);
- Knowledge mobilisation – shared data-sets, best practice, multidisciplinary partnerships;
- Models of pollution-related harms: e.g. *in vivo*, *in vitro*, *in silico* for establishing levels of exposure and exploring biological causation pathways in whole systems versus specific mechanisms;
- Causally informed Mendelian randomisation studies;
- Interdisciplinary collaboration and communication between exposure scientists, health professionals, toxicologists, government and industry;
- Assessing co-exposures – interactions between distinct air pollutants and the resulting effects on the viability, allergenicity, toxicity and pathogenicity;
- What core measurements are required: what, in how much detail, for how long and for what purpose(s)?
- More longitudinal cohorts are needed.

## Appendix 3

Measures of outcome for specific medical conditions.

- These may include risk phenotypes of conditions of interest (e.g. low mood, psychosis experiences, ischaemic heart disease, asthma in childhood).
- Outcomes of mental health effects: individually, in combination with medical conditions; data including the timing of one or the other condition suitable for longitudinal data analyses.
- Related and relevant demographic (age/gender/ethnicity), and psychosocial variables (social support, relationships, emotional dysregulation, adversity, poverty, trauma, poorer places and environments).
- Exploring which biopsychosocial and eco-social and bio-bio interfaces are relevant for creating disease vulnerability to bioparticles, as well as which psychosocial variables lead to greater exposure: poorer places have poorer housing, poorer food outlets, more crime, violence, less safety, smaller houses, less green space). What are the types of interactions between these? To what extent are gene×environment interactions relevant versus direct toxic effects?
- Which poor health outcomes resulting from poor air quality can lead to poor mental health: e.g. respiratory disease or obesity being associated with depression?
- Multimorbidity tends to occur in poorer places and is driven by both biological vulnerability and psychosocial adversity over the life course and in contemporary environments; how does air quality interact with this causal web of poor health and premature mortality for a subset of the population who are most likely to be exposed to poor air quality?
- What is the role of inflammation and oxidative stress?
